# TP53 in Myelodysplastic Syndromes

**DOI:** 10.3390/cancers13215392

**Published:** 2021-10-27

**Authors:** Yan Jiang, Su-Jun Gao, Benoit Soubise, Nathalie Douet-Guilbert, Zi-Ling Liu, Marie-Bérengère Troadec

**Affiliations:** 1 Department of Hematology, The First Hospital of Jilin University, Changchun 130021, China; jiangyanjdyy@jlu.edu.cn (Y.J.); sjgao@jlu.edu.cn (S.-J.G.); 2 Univ Brest, Inserm, EFS, UMR 1078, GGB, F-29200 Brest, France; benoit.soubise@hotmail.fr (B.S.); nathalie.douet-guilbert@chu-brest.fr (N.D.-G.); 3 CHRU Brest, Service de Génétique, Laboratoire de Génétique Chromosomique, F-29200 Brest, France; 4 Cancer Center, The First Hospital of Jilin University, Changchun 130021, China

**Keywords:** *TP53*, myelodysplastic syndromes, mutation, monoallelic, biallelic, target therapy, MDM2

## Abstract

**Simple Summary:**

The importance of gene variants in the prognosis of myelodysplastic syndromes (MDSs) has been repeatedly reported in recent years. Especially, *TP53* mutations are independently associated with a higher risk category, resistance to conventional therapies, rapid transformation to leukemia, and a poor outcome. In the review, we discuss the features of monoallelic and biallelic *TP53* mutations within MDS, the carcinogenic mechanisms, and the predictive value of *TP53* variants in current standard treatments including hypomethylating agents, allogeneic hematopoietic stem cell transplantation, and lenalidomide, as well as the latest progress in TP53-targeted therapy strategies in MDS.

**Abstract:**

Myelodysplastic syndromes (MDSs) are heterogeneous for their morphology, clinical characteristics, survival of patients, and evolution to acute myeloid leukemia. Different prognostic scoring systems including the International Prognostic Scoring System (IPSS), the Revised IPSS, the WHO Typed Prognostic Scoring System, and the Lower-Risk Prognostic Scoring System have been introduced for categorizing the highly variable clinical outcomes. However, not considered by current MDS prognosis classification systems, gene variants have been identified for their contribution to the clinical heterogeneity of the disease and their impact on the prognosis. Notably, *TP53* mutation is independently associated with a higher risk category, resistance to conventional therapies, rapid transformation to leukemia, and a poor outcome. Herein, we discuss the features of monoallelic and biallelic *TP53* mutations within MDS, their corresponding carcinogenic mechanisms, their predictive value in current standard treatments including hypomethylating agents, allogeneic hematopoietic stem cell transplantation, and lenalidomide, together with the latest progress in TP53-targeted therapy strategies, especially MDS clinical trial data.

## 1. Introduction

Since its identification in 1979 and the revelation of its role as a tumor suppressor gene in 1989, *TP53* (also known as p53) has always been a shining star in the field of cancer research [1,2]. In the past 40 years, researchers have described the protein structure, functions, and regulation mechanism of wild-type TP53. The usage of alternate promoters and alternative splicing of introns result in multiple transcript variants in a tissue-dependent manner. Meanwhile, as the most frequently mutated gene, the frequency of *TP53* mutations is highly variable in different types and different stages of cancers. In addition, the dominant-negative (DN) and gain-of-function (GOF) effects are associated with specific mutations. Hence, ascertaining the tumorigenic mechanism of TP53 is essential for developing a precise treatment regimen in specific tumors [3].

Myelodysplastic syndromes (MDSs) are a group of acquired clonal stem cell disorders, which are very heterogeneous for their morphology, clinical features, survival of patients, and evolution to acute myeloid leukemia (AML). Several prognostic scoring systems including the International Prognostic Scoring System (IPSS), the WHO Typed Prognostic Scoring System (WPSS), the Revised-IPSS (IPSS-R), and the Lower-Risk Prognostic Scoring System (LR-PSS) have been introduced for categorizing the highly variable clinical outcomes (based on survival time and time to AML evolution) within each subgroup; the predictive value of them were validated in a number of independent studies [4,5,6,7,8]. Currently, all the most widely used MDS prognostic scoring systems score bone marrow (BM) blast percentage, depth of cytopenia (hemoglobin, platelet, and absolute neutrophil count), and cytogenetics, and these three major features have been proven to have significant effects on the survival and risk of AML transformation. Other parameters, such as age, lactate dehydrogenase (LDH), ferritin, beta-2 microglobulin, BM fibrosis, and performance status, were also shown to have a less significant prognostic impact on MDS [9]. However, as for all classification systems, variables outside the current parameters prompt further refinements in prognostic scoring systems, in which gene variants have been identified in recent studies, contributing to the clinical heterogeneity of the disease course and influencing the prognosis of patients [10,11]. Notably, *TP53* mutations, with an overall incidence of about 10% in *de novo* MDS and 40% in therapy-related MDS (t-MDS patients) [12,13], is independently associated with resistance to conventional therapies, rapid transformation to AML, and a poor outcome [14]. Of note, in MDS cases with five or more karyotype abnormalities, the absence of *TP53* mutations is associated with a much better survival compared to those with *TP53* mutations [15].

Herein, we discuss the features of *TP53* mutations within MDS, their corresponding carcinogenic mechanisms, their predictive value in current standard treatments including hypomethylating agents (HMAs), allogeneic hematopoietic stem cell transplantation (HSCT), and lenalidomide, together with the latest progress in TP53-targeted therapy strategies, especially MDS clinical trial data.

## 2. Brief Presentation of Wild-Type TP53

### 2.1. TP53 Gene and mRNA

The *TP53* gene is located on the short arm of chromosome 17 (band 17p13.1); it spans 20kb and contains 11 exons and two cryptic exons (9β and 9γ) [16,17]. Multiple transcript isoforms expressed in a tissue-dependent manner result from alternative splicing of this gene and the use of alternate promoters. So far, nine *TP53* mRNAs encoding 12 different TP53 protein isoforms have been reported in humans. They result from the usage of two alternative promoters (the proximal promoter P1 and the internal promoter P2) and alternative splicing of intron 2 and intron 9. The translation is initiated at codon 1 or 40 for mRNAs transcribed from P1. Translation starts at codon 133 or 160 for mRNAs transcribed from P2. Hence, the isoforms are named as p53α, p53β, p53γ, Δ40p53α, Δ40p53β, Δ40p53γ, Δ133p53α, Δ133p53β, Δ133p53γ, Δ160p53α, Δ160p53β, and Δ160p53γ, among which, p53α (also known as canonical TP53) is the fully spliced *TP53* transcript encoding 393 amino acids. Normally, several TP53 isoforms are concomitantly co-expressed in a tissue-specific way, and none of the TP53 isoforms, including canonical p53α, can annihilate the expression or activity of the other co-expressed TP53 isoforms. Therefore, the cell response mediated by TP53 results from the cumulative effect of all the TP53 isoforms expressed within the same cell. The balance of expression among different TP53 isoforms is crucial in predicting cell fate outcome [17]. To date, the precise level of expression, tissue distribution, and biological function of each isoform is still poorly understood [18].

### 2.2. The Full-Length Structure of TP53 Protein and the Major Functions within Each Domain

The canonical TP53 protein contains an acidic N-terminus transactivation domain (TAD) (amino acids 1–62), a proline-rich domain (PRD) (amino acids 63–94), a central DNA-binding domain (DBD) (residues 94–292), a tetramerization domain (TD) (amino acids 325–356), and a carboxy-terminal negative regulatory domain (CTD) (amino acids 356–393) [19,20]. The TAD interacts with regulatory factors, including the negative regulator MDM2 and histone acetyltransferase CBP/p300. The PRD plays a role in TP53 instability and TP53-mediated apoptosis [21]. The DBD binds specifically to double-strand target DNAs and performs cell cycle arrest and tumor suppression function [22]. The highly conserved features within this domain during evolution indicates its importance in TP53 function [23]. The TD regulates the oligomerization state and modulates thermodynamic stability of TP53, and it is also required for the gain of function by TP53 mutants [24]. The CTD is a domain with negative regulatory function, and participates to induce cell death [20].

### 2.3. The Function and Regulation Mechanism of TP53

The TP53 protein is produced in the cytoplasm. It can then be transported into the nucleus where it plays its tumor-suppression role, in particular for prevention of inappropriate cell proliferation and maintenance of genome integrity after genotoxic stress. In some cases, TP53 can be excluded from the nucleus as a result of cytoplasmic sequestration, or hyperactive nuclear export; the aberrant retention of TP53 in the cytoplasm is less responsive to genotoxic stress. In addition, a portion of TP53 localized in the mitochondria induces apoptosis in a transcription-independent manner [25]. In response to stress, TP53 is post-translationally modified by acetylation, phosphorylation, ADP-ribosylation, ubiquitylation, sumoylation, neddylation, and cytoplasmic sequestration. Those modifications participate in its stabilization and nuclear accumulation, and activation as a transcription factor. Activated TP53 functions in a tetramer conformation and promotes the transcription of hundreds of downstream target genes to exert its tumor suppression effectiveness. For example, when the nuclear DNA is damaged by intracellular and/or extracellular stimuli (e.g., toxic chemicals, infectious virus, ionizing or UV radiations, heat shock, hypoxia, or oncogene overexpression), TP53 will enter and accumulate in the nucleus, bind directly to the DNA, and determine the fate of the cell [20]. During this process, the MRE11-RAD50-NBS1 (MRN) complex acts as the sensor of DNA damage: RAD50 recognizes the DNA, NBS1 recruits other DNA repair proteins to double-strand break lesions, and MRE11 processes the DNA ends with its DNA nuclease activity; altogether this activates Ataxia Telangiectasia Mutated (ATM). Activated ATM initiates the TP53-MDM2 oscillator to produce TP53 pulses. In the feedback loop, activated ATM induces a conversion of TP53 from the inactive state to the active state by phosphorylation on serine 15, then active TP53 promotes production of MDM2 in the cytoplasm, which then enters the nucleus to induce degradation of active TP53. Meanwhile, cytoplasmic MDM2 promotes the translation of TP53 mRNA to produce inactive TP53. The number of TP53 pulses induced by the oscillator depending on the extent of DNA damage determines the cell fate. If there are low levels of DNA damage, few TP53 pulses induce transient cell cycle arrest and recruit other proteins to fix the damage. If there are high levels of DNA damage, sustained TP53 pulses suppress DNA repair and induce apoptosis [26,27]. Similarly, cytoplasmic TP53 is transported into the mitochondria under stress conditions and interacts with pro- and anti-apoptotic members of the B cell lymphoma 2 (BCL2) family. This triggers the release of mitochondrial factors driving apoptosis or autophagy [28]. Through the ways described above, TP53 prevents DNA damage accumulation in cells, which could lead to the formation of tumors if such cells continue to divide in an uncontrolled way.

Naturally, in unstressed cells, a low level of short-lived TP53 is expressed, localized in both the nucleus and cytoplasm, and is mainly maintained in an inactive form through transcriptional inhibition; it is usually undetectable by standard immunohistochemistry and immunocytochemistry. However, the half-life of TP53 can be rapidly prolonged from minutes to hours under stress [20,29]. Once the stress is resolved, TP53 can be proteasomally degraded by a number of E3 ubiquitin ligases. Among which, the ubiquitously expressed proto-oncogene MDM2 and its close homolog MDMX are the major E3 ubiquitin ligases involved in this process, and they are critical for regulating TP53 homeostasis. In addition, TP53 can be continuously degraded by the 20S core catalytic chamber of the proteasome, which is independent of ubiquitin. The constant cycle between production and then degradation of TP53 is pivotal for rapid response to oncogenic stress signals, which ensure cell growth under normal conditions [30].

## 3. The Anomalous Function of Mutant TP53

**Figure 1:**
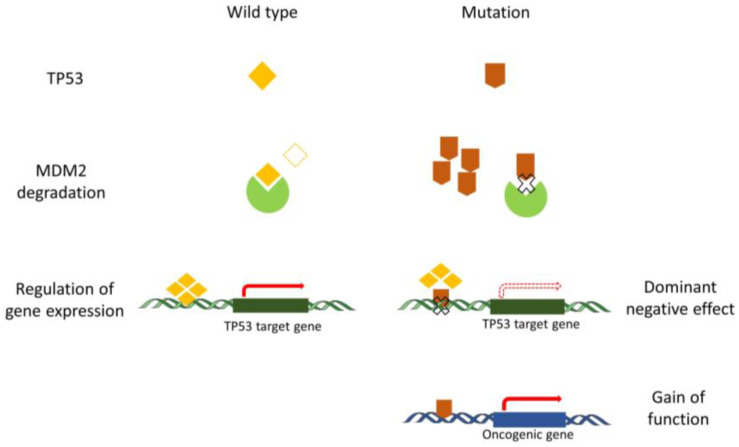
The anomalous function of mutant TP53 compared to the wild-type TP53. Regular degradation of TP53 is fine-tuned in the cell in order to modulate the steady-state level of TP53. Degradation of wild-type TP53 is orchestrated by MDM2 and controls the level of activation of different TP53 target genes. Mutant TP53 proteins are not appropriately degraded by MDM2 and exert a dominant negative effect by preventing wild-type P53 from binding to the regulatory regions of TP53 target genes. Mutant TP53 proteins also play a gain-of-function role that is considered oncogenic.

Normally, the quantity and active state of wild-type TP53 protein can be precisely regulated following an appropriate stimulus. Following the stimulus, cells sense a state of ‘relative’ TP53 deficiency and reduce TP53 degradation. When the state of TP53 deficiency results from a mutation, the TP53 mutant is not able to regulate the downstream genes, and the cells still reduce TP53 degradation. Importantly, the stabilization of TP53 protein is a major hallmark of loss of function secondary to a mutation in the absence of a stimulus. The increase in TP53 mutant levels does not restore wild-type TP53 function, resulting in a limitation of TP53 degradation. Consequently, no additional inhibition of degradation is possible after DNA damage in TP53 mutant cells [31]. In addition, TP53 functions as the transcription factor on hundreds of downstream genes through a tetramer conformation, TP53 mutant proteins can heterodimerize with wild-type TP53 proteins, and the tetrameric complex causes conformational shifts or inhibits the DNA-binding activity of wild-type TP53. While, post-translational modifications and protein-protein interactions alter TP53 tetramerization, then affect transcription, stability, and localization of TP53 [32,33]. Aside from the DN effects described above, GOF functions for some mutations in TP53 are also reported. In those cases, TP53 behaves rather as an oncogene [34,35]. Due to the difference in the biological functions for each TP53 mutant, the mechanisms of GOF still remain poorly described. Currently, results indicate that the oncogenic function of TP53 mutant is mainly caused by the alteration of the binding between TP53 mutant and other oncogenic or tumor suppressive proteins. The stabilization of mutant TP53 is crucial for its GOF activity [36] (Figure 1).

## 4. *TP53* Mutation Features within MDS

### 4.1. Hotspot Missense Mutation on DBD in Diverse Cancers and MDS

Frameshift or nonsense mutations commonly inactivate tumor suppressors. However, an analysis of 24,785 *TP53* mutations in human cancers showed that 73.4% of *TP53* mutations are missense mutations, while nonsense only represent 7.67% and frameshift represent 9.02% [37]. Especially in MDS patients, 78% of missense, 7% of nonsense, 9% of frameshift insertions or deletions, and 5% of splice-site mutations were detected among a total of 396 *T53* mutations [38]. The frequency of each major *TP53* mutation type in MDS is consistent with the counterpart in total human cancers. Furthermore, 86% of *TP53* mutations cluster mainly in the DBD, which is the most highly conserved region among species, and most mutations in this domain are missense (87.9%). In contrast, missense mutations represent only about 40% outside the DBD domain; most mutations are frameshift or nonsense [37]. A crystal structure of a TP53-DNA complex containing the DBD of human TP53 and a DNA binding site helps us to understand why some special sites on DBD are the hotspots of missense mutations. The TP53 DBD serves as a scaffold for two large loops and a loop-sheet-helix motif. A zinc atom keeps the two loops together by tetrahedral links. The DNA binding surface of TP53 is formed by the loop-sheet-helix motif. Hotspot mutations are located at residues implicated either in contact with DNA or in the structure of the DBD [39]. Consequently, the mutations R273C/H, R248Q/W, and R282W are grouped as contact mutations, and render TP53 incompetent for DNA binding. The mutations R175H, Y220C, and G245S are grouped as structural mutations, characterized by the lower thermodynamic stability and unfolded structure compared to the wild-type TP53 [40]. Loss of sequence-specific DNA binding activity caused by hotspot mutations on DBD is a critical reason for TP53 inactivation.

The location and nucleotide substitution of the mutations as well as its frequencies possess histological specificity. For example, the variant R175H is the most frequent missense mutation observed in colorectal cancer, esophagus and stomach adenocarcinoma, breast cancer, and lymphoid leukemia. The variant R248Q is most frequent in oral squamous cell carcinoma (SCC), bladder transitional cell carcinoma, cervix cancer, and myeloid leukemia. C176F is specific to esophagus SCC, R249S is specific to hepatocellular carcinoma, and H179Y is dominant in skin basal cell carcinoma [41]. Each mutation has a specific impact on survival. In high-grade serous ovarian cancer, for instance, G266, Y163C, and R282, when together, are associated with a worsened overall and recurrence-free survival (RFS) compared with other hotspot mutations [42]. The association of specific hotspot mutations with different protein expression patterns followed with different functions may account for the discrepancy of prognosis. Focusing on the spectrum of *TP53* mutations makes it possible to identify mutation patterns associated with etiology, cancer type, therapeutic response, and even target drug selection.

**Figure 2:**
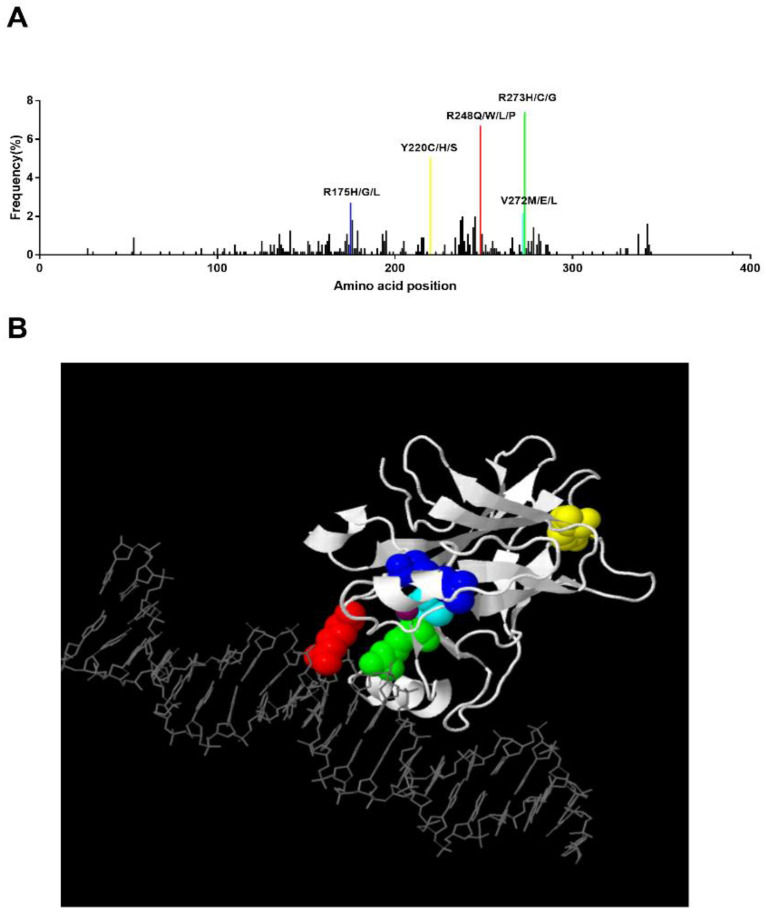
Hotspot TP53 mutations in MDS and the corresponding 3-D structure. (**A**) Distribution of identified TP53 mutations in MDS. The frequency of each mutation was calculated upon pooled data extracted from the IARC TP53 Database (2019) and Bernard et al.’s study. Hotspot mutations with a frequency higher than 2% are labeled [14,43]. (**B**). Three-dimensional view of TP53 binding to DNA. This structure was generated by JSmol software in IARC TP53 database. Residue 175: blue, Residue 220: yellow, Residue 272: cyan, Residue 273: green, Residue 248: red. Zinc: purple.

In MDS patients, Montalban-Bravo et al. reported that the most prevalent mutation was R273H (5%) followed by R248W (4%), Y220C (4%), and R175H (3%) [38]. In Bernard et al.’s study, they also revealed that R273H/C/G, R248Q/W/P/L, Y220C/H/S, and R175H/G/L present higher mutational frequencies [14]. We analyzed the *TP53* mutation pattern of 66 MDS patients in WHO’s International Agency for Research on Cancer (IARC) TP53 Database [43], and a similar mutant pattern of R248Q (9.1%), R273H (6.1%), Y220C (6.1%), and R175H (1.5%) was found (Figure 2A). We generated a 3-D structure using the JSmol software in the IARC TP53 database, which highlighted the hotspot residues above (Figure 2B). In this 3-D model, we can clearly see that residues 248 and 273 can directly bind to DNA, while residues 175 and 220 work as core parts of the structure. In addition, correlation of these hotspot mutations and prognosis in MDS has been reported too. The presence of monoallelic mutations at residues R175 and R248 increases the risk of death compared to non-mutated patients [14]. In vivo proof has demonstrated that R175H and R248Q perform an oncogenic GOF in leukemogenesis, which promotes chemoresistance, invasiveness, and an epithelial-to-mesenchymal transition through diverse mechanisms [44,45].

### 4.2. Biallelic TP53 Dysfunction Predicts Poor Prognosis in MDS

MDS occurs mainly in the elderly. At diagnosis, the median age is ~76 years old in Europe and almost 10 years younger in Asian MDS patients [12]. About 44% of healthy individuals at 50 years of age may have at least one hematopoietic stem/progenitor cell (HSPC) that carries a randomly generated functional *TP53* mutation. Upon exposure to cytotoxic therapy, these mutations can undergo Darwinian selection [46], which may result in the expansion of HSPCs carrying these mutations. However, the high frequency of elderly individuals with heterozygous *TP53* mutations in their circulating leukocytes exceeds the prevalence of MDS or AML in the same age group. Additional mutations and/or chromosomal abnormalities, including mutation or deletion of the second *TP53* allele, are needed for MDS or AML transformation [13].

Donehower et al. carried out an analysis of *TP53* mutations in 32 cancer types and 10,225 patients from The Cancer Genome Atlas (TCGA) and found that more than 91% of cancers with *TP53* mutations show bi-allelic loss of functional *TP53* [47]. Besides, Bernard et al. analyzed 3324 MDS patients with *TP53* mutations, and showed that monoallelic mutations are found in one-third of *TP53*-mutated patients whereas two-thirds present multiple TP53 hits (multi-hit), most of them proven to be biallelic. Excluding *TP53*, driver mutations were more frequent in the monoallelic state compared to the multi-hit *TP53* subgroups. Notably, no identifiable driver mutations other than *TP53* were found in 40% of multi-hit patients, while 90% and 50% of monoallelic patients presented at least one other driver mutation and at least three, respectively. The authors concluded that monoallelic *TP53* mutations are not independently responsible for MDS or AML transformation and predictive of adverse risk. Associations with complex karyotype, few co-occurring mutations, high-risk presentation, and poor outcomes were specific to multi-hit patients, and only multiple TP53 hits was shown to predict the risk of death and leukemic transformation independently of the IPSS-R. Surprisingly, outcomes and response to therapy were not different between monoallelic patients and wild-type *TP53* patients. The co-occurring mutations additional to the monoallelic TP53 mutation shape disease pathogenesis and outcomes [14].

In conclusion, monoallelic *TP53* mutation itself is insufficient to give rise to MDS, and additional other driver mutations or the second *TP53* dysfunction are required to sustain malignancy. Among them, the loss of functional TP53 protein by loss of both *TP53* alleles is associated with a poor outcome for MDS patients with *TP53* mutations.

### 4.3. TP53 Mutations Are Associated with Higher Risk Cytogenetics in MDS

MDS patients carrying *TP53* mutations present a higher frequency of karyotype abnormalities compared to patients without the *TP53* mutation [48]. Among these, abnormal karyotypes; monosomal karyotype, such as -5 and -7, i(17q)/17p-, 5q-; and complex karyotypes possess notable associations with *TP53* mutations [13,15,48,49,50,51]. According to the New Comprehensive Cytogenetic Scoring System for MDS, -7 and complex karyotypes were categorized into poor and very poor prognosis subgroups, which indicate a shorter overall survival (OS) and higher risk of AML transformation [52]. On the contrary, an absence of *TP53* mutations in high complex karyotype (>4 chromosomal aberrations) is associated with an IPSS-R intermediate-like risk [15]. i(17q) and 17p- accompanied by a TP53 mutation results in a loss of heterozygosity (LOH) of TP53. The 5q deletion detected in MDS patients with *TP53* mutations, which more frequently presented a larger deletion size [53], were associated with clonal evolution into complex karyotypes [51,54] and an increased risk of leukemic evolution [55]. The treatment of lenalidomide in MDS patients with isolated del(5q) harboring *TP53* mutations is at a high risk of treatment failure and disease progression; this heterogeneity caused by *TP53* mutations may significantly affect clinical decision making for MDS-del(5q) [56]. The genomic instability caused by TP53 inactivation was considered the pivotal reason for the accumulated chromosomal rearrangements [38,57], and the karyotype complexity is highly related to the variant allele frequency (VAF) of *TP53* [58], which is an independent adverse prognostic factor for OS in MDS patients with *TP53* mutations [59].

### 4.4. The Concurrent Driver Mutations of TP53 Mutation in MDS

Additional driver mutations except for *TP53* are needed for MDS genesis and progression, especially in *TP53* monoallelic patients [14]. Hence, digging out the concurrent mutations is essential for understanding the pathogenesis of MDS. Meanwhile, with the development of next-generation sequencing (NGS) and gene targeting therapy, TP53-targeted therapy in combination with drugs aimed at concurrent driver mutations may be a potential treatment approach in the future. The sequencing spectra of molecular aberrations in the presence of *TP53* mutations were inconsistent in different reports, due to the heterogeneity of the panel of genes used for each study, and the fact that some studies integrated the bi-allelic *TP53* state. Co-occurring somatic mutations were found in 48% of MDS patients harboring *TP53* mutations in Cluzeau et al.’s study, in which *TET2*, *DNMT3A, JAK2, ASXL1, U2AF1, PPM1D, SF3B1,* and *NF1* account for the most frequently concurrent gene mutations, which is similar to Haase et al.’s results [15,60]. Compared to MDS patients without *TP53* mutations, *ASXL1*, *RUNX1*, *U2AF1*, *JAK2*, and *SF3B1* are less detectable in *TP53* mutant cases, in contrast to *TET2*, which is more frequent in *TP53* mutation patients [15]. Analysis of the frequency of driver mutations within the *TP53* state showed that *TET2, SF3B1, JAK2, ASXL1, SRSF2, CBL, RUNX1*, and *BCOR* are significantly higher in monoallelic compared to biallelic *TP53*-mutated patients [14]. Mutations of *TET2* and *TP53* predict poor survival in MDS patients receiving HMTs or HSCT [61,62]. TET-selective small molecule inhibitor suppresses the clonal evolution of *TET2* mutant murine cells, as well as human *TET2*-mutated leukemia xenografts without affecting the normal hematopoietic precursor cells in vitro and in vivo. Indeed, TET2 encodes a methylcytosine dioxygenase essential for myelopoiesis. The survival and proliferation of cells with *TET2* mutations are critically dependent on residual TET activity derived mostly from TET3 and TET1. The TET inhibitor transiently suppresses the residual methylcytosine dioxygenase activity and eventually eliminates the tumor initiating clones with *TET2* mutations [63]. This result suggests that the combination of specific *TET* and *TP53* target drugs could be considered as a new therapeutic strategy for MDS patients presenting these two concurrent mutant genes.

## 5. The Predictive Power of TP53 Mutations in the Current Standard Therapy of MDS

Most of the *TP53*-mutated MDSs belong to the higher risk subtypes, and almost two third are MDS with excess of blasts (MDS-EB) [64,65]. Therapeutic approaches for higher-risk patients aim to change the course of the disease, improving OS, and attempt to postpone disease progression. Currently, allogeneic transplantation remains the only potentially curative option if the patients are eligible, and HMAs are proposed as cytoreduction before transplant and for nontransplant patients [66]. However, accumulated evidence indicates that *TP53* mutations, especially the multi-hit *TP53* mutations, are crucial negative markers for the treatment outcomes of these two key therapeutic strategies [65].

### 5.1. The Impact of TP53 Mutations on Allogeneic-HSCT (a-HSCT)

*TP53* mutations are significantly associated with a higher risk of relapse and shorter progression-free survival (PFS) and OS after transplantation independent of the age and Karnofsky performance status score and IPSS-R [67,68,69,70]. Meanwhile, patients who received reduced intensity conditioning regimens presented similar adverse effects to those who received myeloablative conditioning regimens. This suggests that the escalation of the intensity of the conditioning regimen is ineffective in improving the outcomes of *TP53*-mutated MDS patients [69]. However, controversial results were also reported, in which no significant difference in prognosis was found after transplantation between the MDS with or without *TP53* mutations [71]. Yoshizato et al. compared the prognosis of *TP53*-mutant MDS in combination with complex karyotypes to patients with *TP53* mutations but without complex karyotypes, and showed that the *TP53* mutation-alone patients had a significantly better OS compared to those with both *TP53* mutation and complex karyotypes [72]. The allelic state of *TP53* and co-occurrent additional mutations may be the root of the discrepancy among different studies, as a cohort study enrolling small-sized samples indicated that *TP53* monoallelic hit patients presented favorable survival compared to multi-hit patients following HSCT, but a larger cohort is warranted to verify this finding [14].

### 5.2. The Predictive Power of TP53 Mutations in Hypomethylating Therapy (HMT)

The prediction of the therapeutic response of HMAs in MDS patients by *TP53* mutations is still controversial. *TP53* mutations had no clear significant impact on response or complete response to azacitidine (AZA) according to several studies [73,74,75,76]. However, both 5-day and 10-day courses of decitabine regimens administered at a dose of 20 mg per square meter of body-surface area per day presented a higher response rates in *TP53*-mutated patients compared to patients with wild-type *TP53* [77,78]. The predictive value of *TP53* mutations to the outcomes of HMT in MDS patients was investigated in a meta-analysis including 22 published articles and 2020 participants. This study showed that the presence of *TP53* mutations predicted an increased overall response rate with HMA treatment [79]. However, the duration of response usually lasted less than a year, which was shorter than that of the wild-type patients, and *TP53* mutations were almost always found at times of disease progression after HMT. The short durations of remission were due to incomplete clearance of malignant clones still presenting driver mutations [75,77,78]. Furthermore, the therapeutic response of HMAs was not associated with an improved survival, the median OS of *TP53* mutation patients was merely half of that in the wild-type *TP53* counterparts, and the dismal outcomes of *TP53* mutations cannot be remedied by HMAs [73,75,76,77].

### 5.3. The Impact of TP53 Mutations on Lenalidomide Treatment in MDS-5q-Patients

Lenalidomide, with about a 70% erythroid response rate, sustained independence from transfusion and longer median survival within the responders, and is currently considered the standard frontline treatment strategy for MDS with isolated del(5q) [56,80]. However, a minority of patients within this lower-risk subgroup still do not respond and progress to leukemia, in whom *TP53* mutations prove to be a strong predictor for the poor outcomes [81].

The only genomic differences between lenalidomide-responsive and non-responsive patients were *TP53* mutations, which were also associated with an absence of hematological or complete cytogenetics response [55,82]. Meanwhile, the *TP53* mutations, harbored by the patients that are less sensitive to lenalidomide, are present years before disease progression or acquired over the course of the disease, and prevail during treatment, leading finally to progression. The inherent genomic instability of subclones with *TP53* mutations makes them more probable to acquiring additional molecular alterations, which are considered the root of lenalidomide resistance [55,83,84].

Of particular importance, MDS-del(5q) harbors unique pathological mechanisms owning to the 5q deletion. The ribosomal protein S-14 (*RPS14*) gene, which is located within the commonly deleted region (CDR) of 5q, encodes a component of the 40S ribosomal subunit. Ribosomal insufficiency caused by haploinsufficiency of *RPS14* disrupts ribosome integrity, and triggers degradation of the human homolog of the mouse double minute 2 protein (MDM2), which ultimately stabilizes TP53. Then, TP53 acts as an antagonist of GATA-1, an erythroid-specific transcription factor required for erythroid differentiation and survival. Sustained stabilization of TP53 in erythroid precursors is therefore a crucial effector of the hypoplastic anemia. Suppression of TP53 with dexamethasone or Cenersen, a *TP53* antisense oligonucleotide targeting *TP53* mRNA, results in a reduction of apoptosis and restoration of erythropoiesis in del(5q) MDS progenitors [85]. Given the unique pathological mechanism of MDS-del(5q), the TP53-targeting strategy within this subtype should be comprehensively organized.

## 6. Novel TP53-Targeted Therapy Strategies

**Figure 3:**
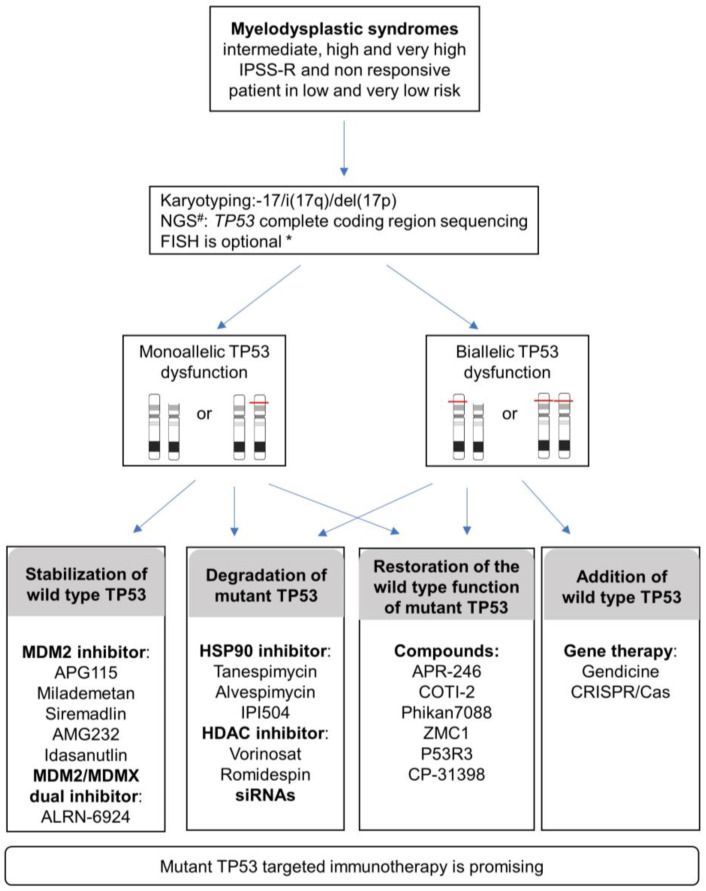
Proposed diagnosis strategies and current TP53-targeting strategies reported in various cancers and that could be applied to MDS patients in IPSS-R intermediate, high, very high risk, and non-responsive patients in very low and low risk. # NGS, Next-Generation Sequencing; * FISH, Fluorescent in situ hybridization. FISH is advised under the following conditions: <20 metaphases or unresolved metaphases or one abnormal metaphase or balanced changes involving breakpoint at 17p13. Specific pipelines of NGS analysis allow assessment of allelic imbalances (gains, deletions, cnLOH) in addition to identifying variants.

The variant allelic frequency burden (VAF) is concordant with outcomes and, more importantly, recent data specifically highlight the importance of the *TP53* allelic state on complexity, prognosis, and clinical presentations. Considering the allelic state of TP53 is thus important for MDS management (Figure 3). The evaluation of the biallelic state is not trivial with routine genetic techniques. In line with Bernard E et al. 2020, a more realistic alternative is to consider multi-hit (rather than bi-allelic) TP53 including multiple mutations, TP53 deletion, and copy neutral loss of heterozygosity (cnLOH). A combination of different techniques can be used to achieve this evaluation, such as conventional chromosome banding (karyotyping) with targeted sequencing of *TP53* exons, FISH, gene panel NGS, or SNP-array. Bernard E et al. 2020 proposed a combination of conventional chromosome banding with NGS because it can also detect allelic imbalance (gains, deletions, cnLOH) when analyzed with specific pipelines.

TP53 evaluation will be particularly valuable for intermediate, high, and very high IPSS-R group of risk MDS and non-responsive patients in very low- and low-risk MDS.

Considering the involvement of monoallelic or biallelic *TP53* together with the functional complexity of *TP53* mutants including loss of function (LOF), dominant-negative (DN), and gain of function (GOF), the current approaches targeting TP53 can be divided into two programs: either restoring the normal TP53 function or abrogating the effect of the anomalous TP53 mutant (Figure 3).

### 6.1. Restoring the Normal TP53 Function

Loss of homologous *TP53* or biallelic inactivation through loss of heterozygosity (LOH) of the remaining normal *TP53* allele can cause a complete loss of function. Apart from aberrations in *TP53* itself, overexpression of the negative regulators of TP53, such as MDM2 and MDMX, can also decrease the TP53 function. Gene therapy and disruption of the binding between TP53 and the downstream negative regulators are the main strategies to restoring the TP53 function.

#### 6.1.1. Adding TP53 or Modifying a Mutant into Wild-Type TP53 by Gene Therapy

Adenovirus gene therapy is the major proposed *TP53* gene therapy strategy till now. Gendicine, approved by the China Food and Drug Administration (CFDA) in 2003 as a first-in-class gene therapy product for the treatment of head and neck cancer, exhibited a significantly higher response rate than standard therapies alone when combined with chemotherapy and/or radiotherapy. Clinical trial data in several other cancer types, including liver cancer, lung cancer, digestive tract cancer, female reproductive cancer, brain cancer, and soft-tissue cancer, yielded favorable progression-free survival [86]. However, no equivalent clinical trial in MDS has been reported yet.

CRISPR/Cas gene editing is a promising technology for treating cancer, with versatile approaches, such as homology-directed repair (HDR), base editing, and prime editing. CRISPR/Cas can potentially target the entire mutated *TP53* locus, replace it with a functional cDNA copy of *TP53* by homologous recombination, or target various hotspot mutations in a more precise way, leading to a single nucleotide substitution or precise gene editing [87]. CRISPR/Cas9 gene-editing therapies for the treatment of hematological diseases including sickle cell disease, β-Thalassemia, leukemia, lymphoma, and multiple myeloma have been tested in clinical trials, and recent results indicate that one patient with sickle cell disease and two patients with thalassemia no longer required blood transfusions [88]. No data for this technique for treating MDS has been reported till now. As the mutation distribution in *TP53* is highly heterogenous and the length of the *TP53* locus is shorter than the limit of the genomic fragment in the CRISPR/Cas technique, replacing the entire TP53 locus would increase the applicability of the CRISPR/Cas therapeutic system for MDS [89]. Furthermore, dCas9 (dead Cas9) fused to transcriptional modulators or histone-modifying enzymes could regulate the TP53 pathway genes. The inherent functions of wild-type TP53 for DNA damage repair may affect the efficacy of CRISPR/Cas9 gene edition. A deeper understanding of the impact of TP53 status on the efficacy of CRISPR/Cas9 machinery will facilitate the development of gene-editing therapies [88]. The CRISPR/Cas targeting therapeutic system continues to evolve to improve accuracy and reduce off-target or side effects. However, accurate and effective vectorization of CRISPR/Cas tools into the targeted cells will also have to be achieved.

#### 6.1.2. Decreasing the Effects of Negative Regulators of TP53 to Stabilize Wild-Type TP53

Human double minute 2 (MDM2) is the key negative regulator of TP53, which can perform ubiquitin-dependent degradation of TP53, and inhibit the transcription regulatory function of TP53 by blocking TAD of TP53 [90]. MDM2 can also export TP53 out of the cell nucleus, preventing TP53 from targeting DNA [91]. Thus, disrupting MDM2 and TP53 interaction ultimately leads to wild-type TP53 stabilization. This is deemed an important step forward in cancer therapy.

Three hydrophobic and aromatic TP53 amino acids (F19, W23, and L26) form a structure that is complementary to and fills up a surface hydrophobic pocket at the N-terminus of MDM2 [90]. This crystal structure provides a framework for the discovery of compounds that may prevent the inactivation of TP53 by MDM2 in cancers.

The understanding of the functions of the nutlins by Vassilev et al. in 2004 and antitumor effect verified in preclinical studies paved the way for molecule inhibitors of the MDM2-TP53 interaction [92]. Following nutlins, several molecules, including: RG7112 (RO5045337), idasanutlin (RG7388), AMG-232 (KRT-232), APG-115, BI-907828, CGM097, siremadlin (HDM201), and milademetan (DS-3032b), have come into clinical practices [93]. Two phase 1 studies of the MDM2 inhibitor AMG-232 or Idasanutlin in combination with or without trametinib or cytarabine for treating AML reported a 31–35.6% response rate in combination groups [94,95]. Clinical investigations of high-risk MDS, such as APG-115 in combination with Azacitidine or Cytarabine (ClinicalTrials.gov identifier: NCT04275518), Milademetan ± 5-Azacitidine (NCT02319369), and Siremadlin in combination with MBG453 or venetoclax (NCT03940352), are ongoing. Furthermore, dual inhibition of MDMX and MDM2 with a stapled α-helical peptide (ALRN-6924) exhibited marked antileukemic effects in vitro and in vivo, which support further testing of the dual inhibitor as a therapeutic approach in cancers with wild-type TP53 [96,97]. It is worth noting that acquired *TP53* mutations emerge during treatment with MDM2 inhibitors, which contribute to the acquired resistance to MDM2 inhibition. The selective pressure induced by the MDM2 inhibitor is considered to be the reason for the rapid emergence of resistance mutations. The combination therapy of mutant TP53-activating drugs with MDM2 inhibitors was proposed to improve the clinical activity of MDM2 inhibitors [98].

### 6.2. Abrogating the Effect of Anomalous Mutant TP53

The TP53 mutant not only loses the tumor suppressor effect, but also affects the normal activity of wild-type TP53 through heterodimerization and gains oncogenic function. Therefore, mutant TP53 targeting has prompted great interest in cancer therapy. Abrogation of the effect of anomalous mutant TP53 can be achieved in two strategic approaches: the first and best is reactivating a wild-type function from a mutant TP53, and the second is the degradation of anomalous mutant TP53.

#### 6.2.1. Wild-Type TP53 Function Reactivation from a TP53 Mutant

Most *TP53* mutations are missense targeting DBD and usually result in one amino acid change. The change destabilizes the DBD folding at physiological temperatures, precluding the proper orientation of loops and helixes present at the DNA-binding interface, or disrupting DNA binding directly by the DNA-binding residue change. It is structurally possible to restore wild-type TP53 functions from TP53 mutant [99]. The proof of concept was done in 1993, when Hupp et al. achieved restoration of the DNA binding of TP53 mutant by modifying phosphorylation or by antibody binding [100]. Subsequently, accumulated investigations from different laboratories have yet to put mutant TP53 reactivation into preclinical and clinical practices.

Several compounds that can reactivate proper TP53 function by converting TP53 mutant into the wild-type conformation or increase the thermal stability of TP53 mutant have been reported [101]. These compounds, including PRIMA-1, APR-246, COTI-2, PK11007, PK7088, Phikan083, Chetomin, NSC319726, P53R3, CP-31398, ZMC1, and 3-AP, can be either specific to certain mutations, such as R175H, R273H, Y220C etc., or more broadly target various mutants of TP53. Among them, APR-246 and COTI-2 present remarkable anticancer activities, and have progressed to clinical trials. Herein, we will present the latest progress for APR-246 clinical trials on MDS.

APR-246 (aka PRIMA-1^MET^ or Eprenetapopt) has received breakthrough therapy, orphan drug, and fast track designations from the FDA for MDS treatment, and orphan drug designation from the European Medicines Agency for MDS. APR-246 is a prodrug that is converted to the active electrophile methylene quinuclidinone (MQ) under physiological conditions, which covalently binds C124 and C277 of TP53 DBD, restores its wild-type conformation, and mediates thermostabilization of the TP53 mutant. In addition, MQ inhibits the selenoprotein thioredoxin reductase 1 and converts it to an active oxidase, depletes glutathione, and induces reactive oxygen species [102]. A recent study also found that APR-246 induces early cell death by ferroptosis [103]. Two studies have evaluated the efficacy and safety of APR-246 in combination with azacitidine in MDS patients with *TP53* mutations (Table 1). HMA-naive MDS patients with higher IPSS-R risk were included in both studies, with more than 85% of the cases carrying complex karyotype and a median *TP53* VAF of 20%. The overall response rate (ORR) was 62 and 73%, and the complete remission rate was 47 and 50%, respectively. In total, 58% achieved a cytogenetic response and 43% yielded a molecular response [60,104,105,106]. From the reported data, the combination of AZA and APR-246 seems to have therapeutic superiority compared to the previously reported single-agent HMAs in MDS patients with *TP53* mutations. The latter represents the standard treatment regimen for higher risk MDS patients at present [107,108]. Maslah et al. found that the synergistic effects of APR-246 with 5-azacitidine in *TP53*-mutated MDS and AML were mediated by downregulation of the FLT3 pathway in drug-treated cells [109]. To investiagte the therapeutic effect, a pivotal phase III, multicenter, randomized study of APR-246 in combination with azacitidine versus azacitidine alone in patients with *TP53*-mutant MDS is ongoing (NCT03745716). Furthermore, another multi-center, open label, phase II clinical trial to assess the safety and efficacy of APR-246 in combination with azacitidine as maintenance therapy after allogeneic HSCT for patients with *TP53* mutant AML or MDS is also ongoing (NCT03931291) (Table 2).

Next, we will present a brief review of PhiKan7088, which targets Y220C, a hotspot *TP53* mutation in MDS. The Y220C mutation is located in the DBD of TP53, which can create a unique surface cavity that destabilizes TP53 protein. The small drugs PhiKan7088 specifically bind to this cavity and interact with Y220C TP53. In vitro, PhiKan7088 corrects the folding of Y220C TP53; restores transactivation abilities; induces growth inhibition, cell-cycle arrest, apoptosis, p21, and NOXA expression; and triggers nuclear export of the pro-apoptotic BAX protein to mitochondria [110]. Research on improving the binding affinity of these small-molecule stabilizers of Y220C TP53 is ongoing [111].

#### 6.2.2. Degradation of Anomalous TP53 Mutant

Increased protein stability of TP53 mutant is involved in several pathways implicated in cancer development, which regulates many cellular processes, including proliferation, survival, invasion, migration, metabolism, chemoresistance, and tissue architecture, to promote tumor progression [112]. TP53 mutants can accumulate to high levels in tumors, which promotes GOF in tumorigenesis [113]. Owing to the GOF effect of mutant TP53, degradation of anomalous mutant TP53 has prompted great interest in cancer therapy. In addition, since the survival advantage conferred by TP53 mutant results in cancer cells being addicted to mutant TP53, anomalous TP53-targeted immunotherapy tends to be a promising anticancer strategy.

Inhibitors targeting the heat shock protein 90 (HSP90) and histone deacetylase 6 (HDAC6) represent viable clinical strategies for anomalous TP53 mutant degradation [114]. The HSP90/HDAC6 chaperone machinery is significantly upregulated in many types of cancers including MDS compared to normal tissues [115], where HDAC regulates HSP90. HSP90 forms stable complex with TP53 mutant, inhibits E3 ligases MDM2 and carboxyl terminus of HSC70-interacting protein (CHIP), stabilizes TP53 mutant, and supports proper folding of TP53 mutant [116]. Then, the TP53 mutant affects the function of wild-type TP53 through a dominant negative effect (refer to Section 3) and members of its family [117].

A few HSP90 inhibitors are currently available, including geldanamycin and a series of derivatives, such as tanespimycin (17-AGG), alvespimycin (17-DMAG), and IPI504. HDAC inhibitors like vorinosat (SAHA) and romidepsin (istodax) have been approved by FDA for T cell lymphoma. HSP90/HDAC inhibitors can indirectly decrease the stability of TP53 mutant, and consequently induce the wild-type TP53 function. Phase I-II clinical trials have evaluated the pharmacokinetics, safety, and efficacy of these inhibitors both as a single agent or in combination with other anticancer medicines for solid tumors, and some hematological malignancies like lymphoma and multiple myeloma [116,118,119,120,121,122,123]. No study on MDS has been reported yet.

Allele-specific small-interfering-RNAs (siRNAs) are specific enough to discriminate mutant from wild-type TP53 transcripts in cells where both wild-type and mutant TP53 are present, silence the expression of the mutant transcripts without affecting wild-type TP53 mRNA, and consequently restore wild-type protein function. This ultimately induces cell death by abrogating both the addiction to mutant TP53 and its DN effect, and retards tumor growth [124,125]. Furthermore, depletion of TP53 mutant mRNA by its specific siRNA was validated in the absence or presence of wild-type TP53. In cancer cells expressing TP53 mutant alone, downregulation of TP53 mutant significantly resulted in decreased cell proliferation and migration. In cancer cells expressing both wild-type TP53 and TP53 mutant, cell proliferation and migration were also reduced. In those cells, loss of the TP53 mutant DN effect and subsequent restoration of wild-type TP53 activity induced the expression of TP53 downstream target genes. A synergistic effect was found after adding an MDM2 inhibitor or a chemotherapeutic agent [126]. Three siRNA therapeutics, patisiran, givosiran, and lumasiran, have been approved by the FDA from 2018 to 2020 for the treatment of peripheral nerve disease secondary to hereditary transthyretin-mediated amyloidosis, acute hepatic porphyria, and primary hyperoxaluria type 1, respectively. With the refinement of N-acetylgalactosamine and the development of other novel strategies for the targeting and delivery of siRNAs, TP53 mutant-specific siRNAs targeting hotspots mutations are sure to follow in the coming years [127].

As cancer cells are addicted to TP53 mutant, anomalous TP53-targeted killing therapy is promising, especially TP53 mutant-targeted immunotherapy. Researchers from Johns Hopkins University School of Medicine described a bispecific single-chain diabody, which can effectively activate T cells to lyse cancer cells that presented a neoantigen derived from the TP53^R175H^ mutation. The intracellular R175H mutant TP53 is degraded into peptides by the proteasome, then a fraction of these peptides, HMTEVVRHC (mutant amino acid underlined), is presented by the human leukocyte antigen (HLA) on the cell surface, making it a neoantigen. The diabody binds bispecifically with high affinity to this neoantigen and the T cell receptor-CD3 complex on T cells, leading to the activation of T cells to secrete cytokines and kill target cancer cells. The precise killing has been confirmed both in vitro and in vivo without affecting the wild-type TP53 counterpart [128].

The precise pathogenetic mechanisms underlying the poor outcomes of MDS patients with *TP53* mutations are still unclear. Sallman et al. described the immunological characteristics of the malignant clone and the alterations of the immune microenvironment in patients with *TP53* mutations and wild-type MDS or sAML. Importantly, the expression of PDL1 is significantly increased and highly immunosuppressive regulatory T cells (Tregs) as well as myeloid-derived suppressor cells are expanded in hematopoietic stem cells of TP53-mutated patients. However, bone marrow-infiltrating OX40+ cytotoxic T cells, helper T cells, and natural killer cells are significantly reduced in cases with *TP53* mutations. The immune privilege and evasive features within *TP53* mutant MDS and the sAML microenvironment may be a primary driver of dismal outcomes. Therefore, immunomodulatory therapeutic strategies may be viewed as beneficial for this molecularly defined subpopulation [129].

## 7. Conclusions

TP53 mutations have been proven to be an adverse marker in the prognosis of MDS patients. They are associated with excess bone marrow blast proportion, thrombocytopenia, complex karyotypes, resistance and early relapse to lenalidomide, HMAs, and aHSCT, all of which result in miserable outcomes for MDS cases with TP53 mutations. Due to the recent findings that the allelic state of TP53 is important for MDS prognosis and management, the evaluation of the biallelic state or, at least, multi-hit TP53 will be relevant in future studies. This evaluation can be done by a combination of karyotyping and NGS. With in-depth research on the pathogenic mechanism of the TP53 mutant and the development of pharmaceutical technology, novel drugs, such as APR-246, may illuminate the future of MDS treatment. Intermediate-, high-, and very high-risk MDS and non-responsive patients in very low- and low-risk MDS would be the first to benefit from these strategies. Given the complexity of TP53 mutations, the implementation of standard diagnostic tests that accurately determine the monoallelic or biallelic status of TP53 is crucial for making tailored therapeutic strategies. MDS could benefit from the different therapeutic approaches that are explored for other cancers, including the stabilization of wild-type TP53, degradation of TP53 mutant, restoration of wild-type TP53 from mutant TP53, and addition of wild-type TP53 (Figure 3). The development of more highly effective TP53 target medicines with fewer side effects is still a challenge.

## Figures and Tables

**Table 1:** Two studies evaluating the efficacy and safety of APR-246 in combination with azacitidine in MDS patients with TP53 mutations.

Reference	Phase NCT	Case	IPSS-R Risk Stratification	Karyotype	TP53 Mutation per Patient Median (Range)	TP53 VAF % Median (Range)	Intervention	Response Rate	Duration of CR, Months, Median (95% CI)	Median OS (Months)
Sallman et al. JCO, 2021 [104]	Ib/II NCT03072043	40	intermediate: 10%, high: 20%, very high: 70%	Complex 90%	1 (1–3)	20 (1–72)	APR-246 (50, 75, 100 mg/kg/d IV(day-14—-11) or APR-246(4500 mg/d) (days 1–4) + AZA 75 mg/m^2^/d (day 4–10 or 4–5 and 8–12), 28 days/cycle	ORR 73%, CR 50%, CGR 58%	7.3 [5.8 to NE]	10.4 (7.6–13.3)
Cluzeau et al. JCO, 2021 [60]	II NCT03588078	34	intermediate: 12%, high: 15%, very high: 74%	Complex 85%, monosomal 79%	1 (1–3)	20 (0.1–83)	APR-246 4500 mg /d IV(days 1–4) + AZA 75 mg/m²/d (days 4–10), 28 days/cycle	ORR 62%, CR 47%	11.4 (6.5 to 16.8)	12.1

ORR: ORR, overall response rate; CR, complete remission; CGR, cytogenetic response; NE, not evaluable.

**Table 2:** The ongoing clinical trials in TP53 mutant MDS.

Number	Identifier	Title	Case	Intervention
1	NCT03745716	A Phase III Multicenter, Randomized, Open Label Study of APR-246 in Combination with Azacitidine Versus Azacitidine Alone for the Treatment of TP53 Mutant MDS	154	Experimental arm: APR-246 + Azacitidine; Control arm: Azacitidine
2	NCT04638309	Phase 1 Study to Evaluate Safety and Efficacy of APR-548 in Combination with Azacitidine for the Treatment of TP53-Mutant MDS	46	APR-548 monotherapy followed by APR-548 + Azacitidine
3	NCT03931291	Phase II Trial of APR-246 in Combination with Azacitidine as Maintenance Therapy for TP53 Mutated AML or MDS Following Allogeneic Stem Cell Transplant	33	APR-246 + Azacitidine
4	NCT02909972	A Phase 1/1b Open-Label Study to Determine the Safety and Tolerability of ALRN-6924 Alone and in Combination with Cytarabine in Patients with Relapsed/Refractory AML or Advanced MDS With Wild-Type TP53	55	ALRN-6924 alone; or Cytarabine followed by ALRN-6924
5	NCT04358393	A Phase Ib/II Study of APG-115 Alone or in Combination with Azacitidine in Patients with Relapse/Refractory AML, CMML or MDS	69	APG-115 alone; or APG-115 + 5-AZA
6	NCT03940352	A Phase Ib, Multi-arm, Open-label, Study of HDM201 in Combination with MBG453 or Venetoclax in Adult Subjects with AML or High-risk MDS	80	HDM201+MBG453; or HDM201 + Venetoclax
7	NCT03855371	Combination of Decitabine and Arsenic Trioxide to Treat AML/MDS Expressing a Classified Type of Mutant p53 (Phase 1)	5	Decitabine + Arsenic trioxide
8	NCT03377725	Decitabine and Arsenic Trioxide in the Treatment of MDS (Phase 3)	200	Experimental arm: Decitabine + Arsenic trioxide;Control arm: Decitabine alone
9	NCT03772925	A Phase 1 Study of MLN4924 (Pevonedistat) and Belinostat in Relapsed/Refractory AML or MDS	30	Belinostat + Pevonedistat
